# Effect of ^18^F-FDG Uptake Time on Lesion Detectability in PET Imaging of Early-Stage Breast Cancer

**DOI:** 10.18383/j.tom.2015.00151

**Published:** 2015-09

**Authors:** Kristen A. Wangerin, Mark Muzi, Lanell M. Peterson, Hannah M. Linden, Alena Novakova, Finbarr O'Sullivan, Brenda F. Kurland, David A. Mankoff, Paul E. Kinahan

**Affiliations:** Departments of 1 Bioengineering,; 2 Radiology, and; 3 Medicine, University of Washington, Seattle, WA;; 4 Department of Statistics, University of Cork, Cork, Ireland;; 5 Department of Biostatistics, University of Pittsburgh, Pittsburgh, PA; and; 6 Department of Radiology, University of Pennsylvania, Philadelphia, PA

**Keywords:** physics, PET, breast imaging, lesion detectability, virtual clinical trial

## Abstract

Prior reports have suggested that delayed ^18^F-fluorodeoxyglucose positron emission tomography (FDG-PET) oncology imaging can improve the contrast-to-noise ratio (CNR) for known lesions. Our goal was to estimate realistic bounds for lesion detectability for static measurements within 1 to 4 hours between FDG injection and image acquisition. Tumor and normal tissue kinetic model parameters were estimated from dynamic PET studies of patients with early-stage breast cancer. These parameters were used to generate time-activity curves (TACs) for up to 4 hours, for which we assumed both nonreversible and reversible models with different rates of FDG dephosphorylation (*k*_4_). For each pair of tumor and normal tissue TACs, 600 PET sinogram realizations were generated, and images were reconstructed using the ordered subsets expectation maximization reconstruction algorithm. Test statistics for each tumor and normal tissue region of interest were output from the computer model observers and evaluated using a receiver operating characteristic analysis, with the calculated area under the curve (AUC) providing a measure of lesion detectability. For the nonreversible model (*k*_4_ = 0), the AUC increased in 11 of 23 (48%) patients for 1 to 2 hours after the current standard postradiotracer injection imaging window of 1 hour. This improvement was driven by increased tumor/normal tissue contrast before the impact of increased noise that resulted from radiotracer decay began to dominate the imaging signal. As *k*_4_ was increased from 0 to 0.01 min^−1^, the time of maximum detectability shifted earlier, due to decreasing FDG concentration in the tumor lowering the CNR. These results imply that delayed PET imaging may reveal inconspicuous lesions that otherwise would have gone undetected.

## Introduction

Positron emission tomography (PET) is clinically used to determine the spread of breast cancer as well as response to therapy (1). Clinical ^18^F-fluorodeoxyglucose (FDG) PET imaging protocols typically state that images are to be acquired 45 to 60 minutes after radiotracer injection (2, 3). However, it has been shown that FDG uptake in malignant tumors increases for many hours after injection, whereas benign and normal tissue uptake begins to decrease earlier, potentially allowing for better identification of malignant lesions (4–9). Thie et al. (10) discussed the potential for improved lesion detection with PET imaging at different times, stressing that contrast is a time-dependent quantity and concluding that choosing an optimum imaging time is a complicated task. Some studies have investigated dual time-point imaging to exploit this time dependence, comparing lesion standardized uptake values (SUVs) at earlier and later times (11, 12).

The goal of this work was to investigate whether and when there is an improved imaging time point for a single static PET scan. Instead of discriminating between benign and malignant lesions, we focused on what imaging time might improve lesion detection, particularly for lesions with low contrast on the threshold of detectability. We hypothesized that imaging at a later time point than current clinical practice, such as 2 or 3 hours after injection, can improve the contrast-to-noise ratio (CNR). We tested our hypothesis in a virtual clinical trial, combining measured dynamic PET data with kinetic modeling and PET data-generation simulations. We reconstructed images using a fully 3D ordered subsets expectation maximization (OSEM) reconstruction algorithm. We then applied model observers with a specific detection task to the static images to determine the time point of maximum lesion conspicuity.

## Methodology

### Patient Data

The University of Washington institutional review board approved the prospective study that acquired the patient data used in this study. The data were acquired between April 2010 and November 2013 using a GE Discovery STE scanner (GE Healthcare) and comprised 60-minute dynamic PET data sets from 23 patients with ER+/HER2− grade 1 and 2 early-stage breast cancer lesions before receiving therapy. Two patients had a second lesion in the contralateral breast. Patient characteristics are shown in Table 1. The injected FDG doses ranged from 222 to 370 MBq. Lesion size was measured by ultrasound or mammography.

**Table 1.** Patient Characteristics (n = 23)

Characteristic	Mean Value	Range
Age (y)	61.7	51.6-80.0
Weight (kg)	80.9	43.6-141.8
Injected dose (MBq)	315	230-366
Blood glucose (mg/dL)	101.5	82-125
Tumor diameter (cm)	1.6	0.6-3.7
Tumor grade	1.6	1-2
Biopsy ER (Allred score)	8	7-8
Biopsy PgR (Allred score)	5.9	0-8
Ki-67 (% staining)	19.5	5-70

Abbreviations: ER, estrogen receptor; PgR, progesterone receptor.

**Figure 1.**
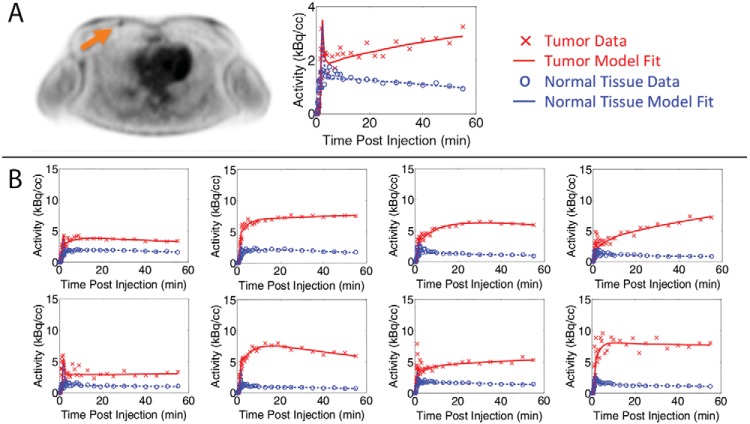
(A) Reconstructed patient image along with measured and modeled decay-corrected TACs for patient 11 with a grade 1, 1-cm-diameter tumor. The acquired dynamic data are shown as data points, and the TACs generated using the parameters estimated with the kinetic model are shown as lines. (B) TAC and model curves for representative patients. Data are plotted based on the middle time point of the time bin.

Images were reconstructed from sinogram data acquired using varying time-bin durations after injection, ranging from 5-second time intervals initially to 5-minute time intervals at 25 to 60 minutes. The OSEM algorithm used 28 subsets and 6 iterations. Image voxel size was 3.3 × 4.3 × 4.3 mm^3^. An example reconstructed image for patient 11 with a grade 1, 1-cm-sized tumor is shown in Figure 1A. Both the tumor and normal breast tissue regions of interest (ROIs) were 3 × 3 × 3 voxels, and the tumor voxel with the highest FDG accumulation was included within the ROI. The tumor FDG uptake values were not corrected for any bias that resulted from partial volume effects. The normal breast tissue ROI was placed in the most homogeneous portion of continuous tissue in the contralateral breast. The tumor and normal tissue time-activity curves (TACs) show the mean FDG uptake in the ROIs as a function of time. The measured decay-corrected TACs for patient 11 are shown in Figure 1A and for a subset of all patients in Figure 1B. One patient with breast implants was excluded from this virtual study because of an abnormally high FDG uptake in the selected normal tissue. Another patient with a tumor with high FDG uptake (>40 kBq/cc) was also excluded because it was outside a relevant range for this detectability study. The final number of patient tumors included in this study was 23.

### Kinetic Modeling

**Figure 2.**
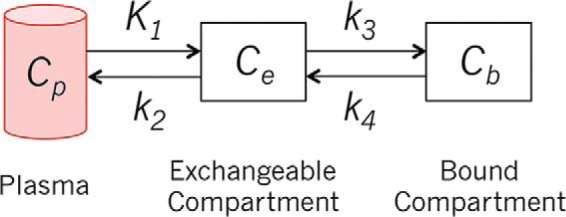
Two-tissue compartment kinetic model.

We used a 2-tissue compartment model (Figure 2) (13) and PMOD kinetic modeling software (PMOD Technologies Ltd.) (14) to estimate the model parameters. The parameters were estimated by matching the model output to the TACs through nonlinear optimization. Patient-specific arterial input functions (AIFs) were extracted from the dynamic time course (15, 16) and then scaled using the activity concentration measured from a left ventricular ROI drawn over the heart. The vascular blood fraction of tissue was fixed at 0.04 mL/mg based on reported experimental measurements of human breast and normal tissues (17, 18). The optimized model TACs are also shown in Figure 1. The estimated model parameters are summarized in Table 2.

**Table 2.** Estimated Kinetic Parameters for All Patients for Nonreversible Model (*k*_4_ = 0)

Parameter	Mean ± SD	Range
*K*_1_ (mL/g/min)	0.009 ± 0.008	0.001–0.019
*k*_2_ (min^−1^)	0.043 ± 0.023	0.005–0.067
*k*_3_ (min^−1^)	0.002 ± 0.003	0.0001–0.006

The estimated kinetic parameters were used to generate simulated TACs up to 4 hours after radiotracer injection. For the forward modeling of the simulated curves, we used an averaged AIF obtained from arterial blood sampling of 13 patients over 5.8 hours (19). The AIF was scaled so that the values at 60 minutes matched those of the patient-specific AIF at 60 minutes. To simulate biological variability, we generated 600 TAC realizations by adding a 10% coefficient of variation to kinetic parameter *K*_1_, which estimates the rate of uptake of FDG into the exchangeable tissue compartment.

We first assumed that *k*_**4**_, the dephosphorylation rate constant of FDG-6-phosphate (FDG-6-P), was 0. This is supported by evidence that FDG becomes trapped in the tumor and is only slowly dephosphorylated because of the low concentration of glucose-6-phosphatase in most cancers, with some notable exceptions such as hepatocellular carcinoma (20). In a number of prior studies that acquired TAC data for up to 1 or 2 hours after radiotracer injection, FDG metabolism was assumed to be irreversible, and *k*_**4**_ was assumed to be 0 (7, 21–24). However, because dephosphorylation has a greater impact on uptake curves at later time points, we investigated several cases of *k*_4_ that were greater than 0. Following published values in the literature (21, 25), we assessed the effect of *k*_4_ values of 0.001, 0.005, and 0.01 min^−1^ on lesion detectability at later time points.

### Image Data Generation

Simulated images were generated for 5 time points (0.5, 1, 2, 3, and 4 hours), with tumor contrasts determined by the synthetic TACs. Tumor and normal tissue FDG activity values were input into a modified National Electrical Manufacturers Association Image Quality phantom (26). Each simulated phantom contained 6 spherical lesions at known locations in separate axial slices. The simulations were to be based on measured patient data. However, the tumors in the patient data were all readily detectable. To generate lesions at the limit of detectability, we simulated the measured radiotracer uptake in 5-mm-diameter tumors for all patients. ASIM, an analytical PET simulator (27), was used to generate emission data (sinograms) based on the detector geometry of a GE Discovery STE PET/CT scanner.

Each noise-free sinogram from ASIM was mapped to a Poisson random sinogram based on the expected number of detected radiotracer decay events, including scattered photons and random coincidences. The ratio of true/scatter/random coincident events was 1:1:0.35. The number of detected events for acquisitions in the first hour after injection was matched to patient data following a 370-MBq injection. For a 5-minute bed position 30 and 60 minutes after injection, 500 and 350 million events would be acquired, respectively. After the first hour, the number of detected events was scaled by the proportionate decrease in normal tissue FDG activity relative to the value at 60 minutes. In this way, we included the effects of radioactive decay and changing activity concentration (as a result of biological decay or washout) in the field of view while ignoring the small contribution of changing tumor uptake to the total number of detected events.

### Image Reconstruction

Images were reconstructed using OSEM with 2 iterations and 32 subsets and postfiltered with a 6-mm full-width half-maximum Gaussian filter. The results shown in this work are after 2 iterations. The image voxel size was 3.3 × 4.3 × 4.3 mm^3^.

### Model Observers

We used both a nonprewhitening matched filter (NPWMF) and a channelized Hotelling observer (CHO) model to estimate lesion detectability (28, 29). Mathematical model observers were used as a surrogate for human observers. Whereas the NPWMF uses a simple matched filter, the CHO model uses spatial frequency-selective channels to model the human visual system. Our CHO model used 4 nonoverlapping rectangular filters and has been validated against human observers (30). The CHO model template was formed using a 20 × 20 pixel region centered over the tumor on 6 training images from each set of simulation parameters (2760 images total). The template was then compared separately to the tumor and normal tissue patches, outputting a test statistic for each. The 600 tumor and 600 normal tissue test statistics over all simulated cases were used to generate receiver operating characteristic (ROC) curves and calculate the area under the curve (AUC) for acquisition times 0.5 to 4 hours after injection. A summary of all simulation steps and parameters is shown in Table 3.

**Table 3.** Simulation Summary

Parameter	Value
Number of patients	23
Kinetic parameters	*K*_1_, *k*_2_, *k*_3_ estimated; *k*_4_ = 0, 0.001, 0.005, and 0.01 min^−1^
Biological noise	10% CV added to *K*_1_
PET data noise	Poisson noise added to sinogram
Noise realizations	600 per patient
Time points	0.5, 1, 2, 3, and 4 hours after injection
Regions of interest	Tumor and normal tissue

Abbreviations: CV, coefficient of variation; PET, positron emission tomography.

## Results

**Figure 3.**
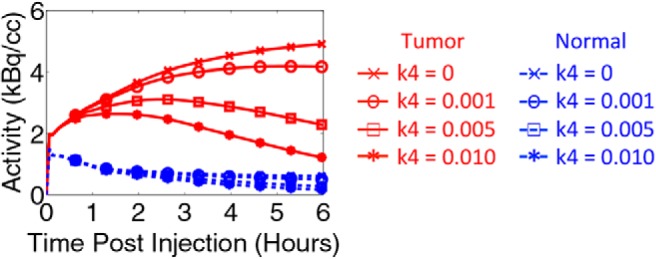
Synthetic tumor and normal tissue TACs for patient 11 assuming *k*_4_ = 0, 0.001, 0.005, and 0.01 min^−1^.

The extrapolated noise-free TACs for patient 11 are shown in Figure 3. In the nonreversible model, the tumor uptake increased for many hours before it began to plateau, which agrees with other reports that suggest that FDG uptake may increase for up to 6 hours before plateauing (4, 6, 7). Normal tissue activity concentration decreased with time. In the reversible model, the impact of nonzero *k*_4_ resulted in a loss of activity from the bound tissue compartment (*C*_m_) and a lower tissue TAC.

**Figure 4.**
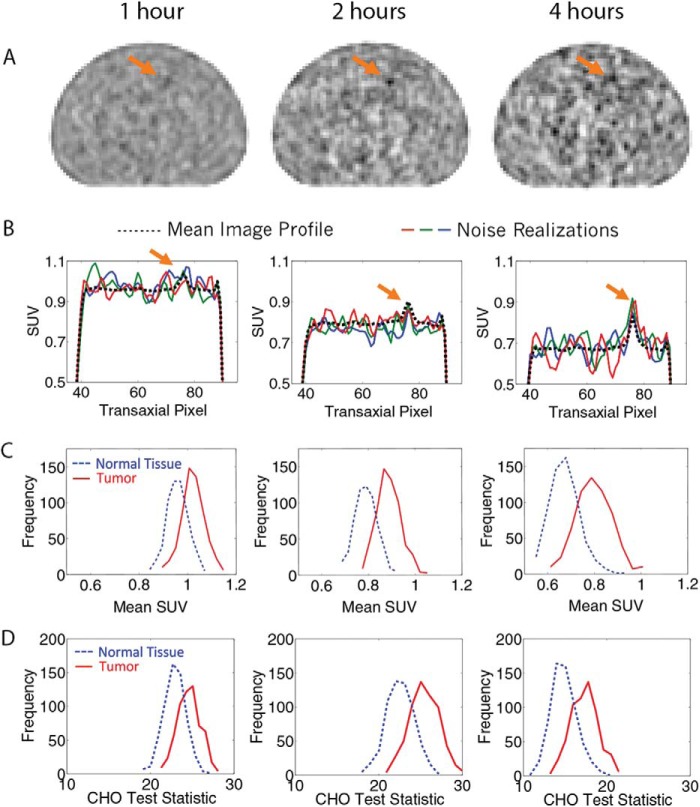
(A) OSEM-reconstructed images for patient 11 and the nonreversible model (*k*_4_ = 0) at 1, 2, and 4 hours after injection; the arrow points to the location of the lesion. (B) Horizontal profiles for 3 noise realizations as well as the mean profile over all realizations. (C) Histograms of normal and tumor tissue SUVs with increasing separation between peaks, with time indicating increasing contrast and a broadening of the distributions indicating increasing noise. (D) Histograms of normal and tumor tissue CHO test statistics—the greater the separation between peaks, the higher the detectability.

Reconstructed OSEM images using the nonreversible model are shown in Figure 4A for patient 11. The images are for 1, 2, and 4 hours after injection and are normalized by the mean. Tumor detectability is visually improved at 2 hours compared with 1 hour; by 4 hours, increases in background noise offset the higher tumor activity. Figure 4B presents horizontal profiles through the tumor for 3 noise realizations as well as the mean profile over all 600 realizations, showing both the increasing contrast and noise with imaging time. Figure 4C shows histograms of the tumor and normal tissue SUVs, calculated as the mean FDG uptake in ROIs that matched the size and shape of the tumor. The normal tissue ROI was selected from a random background location. Figure 4D shows the histograms of the CHO test statistic. The increasing separation of the 2 histograms and consequent tumor detectability resulted from the increased tumor contrast. The increasing width at later time points resulted from increasing image noise caused by radiotracer decay.

**Figure 5.**
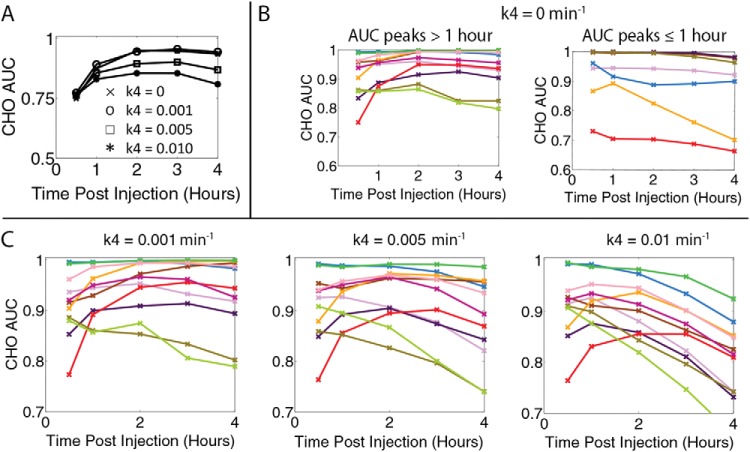
(A) AUC as a function of time calculated from CHO ROC curves for patient 11. (B) AUC results for all patients using the nonreversible model, separated by the AUC peaking after 1 hour (left) or at or before 1 hour (right). Each patient is represented as a different line and color. (C) AUC for the reversible model and patients who benefited from delayed imaging when *k*_4_ = 0. As *k*_4_ increases, the peak AUC, or lesion detectability, shifts earlier.

ROC curves plotting the true versus false-positive fractions were generated from the CHO test statistics for tumor and normal tissue. Figure 5A shows the calculated AUCs from the CHO test statistics for patient 11. The NPWMF observer model AUC trends (data not shown) were similar to those from the CHO model. The AUC results for all patients suggested that they divided into 3 groups. Some tumors showed improvement in detectability with later imaging times, whereas others showed decreasing detectability with increasing times. The third group included the tumors that, despite the small 5-mm size, were always detectable. The results for the first 2 groups are shown in Figure 5B. The curves were divided based on the time of peak AUC; 11 of 23 (48%) of patients' AUCs peaked after 1 hour, and 7 of 23 (30%) of patients' AUCs peaked before or at 1 hour. The remaining 5 patients (22%) had an AUC=1 for all time points, indicating that the tumor was always detectable.

The kinetic modeling simulations and analyses were repeated for the reversible model. The AUC results for patient 11 are also shown in Figure 5A. Figure 5C shows the results for all patients for whom the AUC curve peaked after 1 hour (Figure 5B), with a separate panel for each *k*_4_ value. The AUC decreased with increasing *k*_4_ and with increasing postinjection time, shifting maximum detectability to an earlier time point. Of the 11 patients who benefited from delayed imaging when *k*_4_ was 0, 7 benefited when *k*_4_ was 0.005 min^−1^, and only 2 benefited when *k*_4_ was 0.01 min^−1^.

## Discussion

We have assessed the effect of uptake time on tumor detectability, evaluating both a nonreversible and reversible FDG phosphorylation model. As hypothesized, the SUV and CHO detectability results showed a trade-off between increasing tumor-to-background ratio and increasing image noise caused by radiotracer decay. Higher tumor contrast at later times led to increased separation between the peaks of the SUV and CHO test statistic histograms, whereas increased noise led to broadened width of the histograms. The AUCs from the CHO metrics quantified this trade-off.

This virtual clinical trial used measured patient data in early-stage breast cancer to generate simulated TACs for up to 4 hours after radiotracer injection. In the nonreversible model, it is assumed that FDG becomes trapped in the tumor and cannot be hydrolyzed by the enzyme glucose-6-phosphatase. Whereas hexokinase phosphorylates FDG into FDG-6-P, glucose-6-phosphatase dephosphorylates FDG-6-P back into FDG that can leave the cell. Although this enzyme is found in many organs, it is most active in the liver and kidney (31), and phosphatase activity levels are low in most cancer cells (32). We investigated the effects of imaging time on tumor detectability both with nonreversible and reversible kinetic models because it is likely that the effects of *k*_4_ are non-negligible after the first 1 or 2 hours after injection.

**Figure 6.**
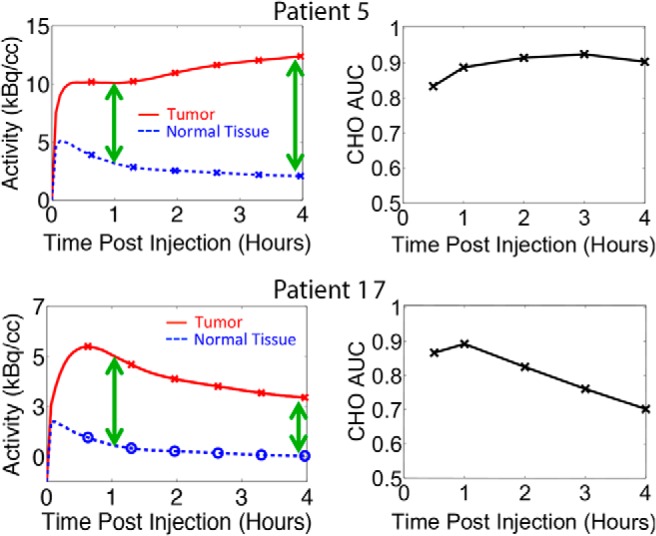
Comparison of patient 5 and 17 TACs to the resulting CHO AUC curves when *k*_4_ = 0. Patient 17 showed the most significant decrease in tumor concentration as well as decrease in AUC as a function of time.

We observed that some tumors were more detectable with delayed imaging with higher AUCs at later times (group 1), whereas others had higher AUCs at the standard uptake time of 60 minutes (group 2). The TAC and AUCs for 2 representative patients are shown in Figure 6. In the first group, the AUCs kept increasing for 2 to 3 hours after injection. The TACs during this time period showed that the tumor FDG concentration increased, whereas the normal tissue FDG concentration decreased. The increasing contrast thus outweighed the increasing noise until later time points when the noise began to dominate and the AUC decreased. In the second group, the AUCs decreased with time because of flat or decreasing tumor TACs that resulted in an unfavorable contrast-to-noise trade-off. Therefore, tumors with low initial contrast and/or a significant increase in contrast with time showed increased detectability with delayed imaging. As *k*_4_ was increased, the concentration of FDG in the tumor decreased more rapidly with time than in the normal tissue for all patients. This trend resulted in decreased tumor contrast, lower AUCs, and thus decreased detectability. With the larger *k*_4_ = 0.01 min^−1^, most AUCs peaked within 1 hour. There was a third group of 5 patients with no detectability trend as a function of imaging time. These patients had tumors with a very large tumor-to-normal ratio, such that the tumors were always detectable (AUC = 1) despite the small tumor size.

The detectability trends depend upon the rates of FDG that accumulate in the tumor, leave the tumor, and clear the blood. The separation of these groups can be predicted by looking at the kinetic rate constants. The overall metabolic flux of FDG into the tumor is calculated as *K*_i_ = *K*_1_*k*_3_/(*k*_2_ + *k*_3_). The mean flux of group 1 was significantly higher than that for group 2 (2-sided *t* test, *P* = .0001). The rate of phosphorylation (*k*_3_) for group 1 was also higher than that for group 2 (*P* = .012). The rate constants also help explain why the FDG concentration peaked early (between 10 and 30 minutes) in 7 of the tumors. These tumors comprised 6 of 7 patients (86%) in group 2, with the remaining patient part of group 3. This early peak could be caused by dephosphorylation (*k*_4_ > 0). Lodge et al. (4) noted that there are other causes of decreasing activity concentrations such as unmetabolized FDG that clear more rapidly from the tissue precursor pool back into the blood (*k*_2_), possibly as a result of a low fixation rate (*k*_3_). Vriens et al. (33) reported that low metabolic tumor regions have a higher blood volume and therefore higher uptake of FDG in the early postinjection time points. With a low phosphorylation rate in these regions, the FDG can clear back into the blood pool.

Our results showed that the detectability trends as a function of time are not uniform and that more information is required to be able to select the optimum imaging time. Ideally, the optimum uptake time for detection could be predicted by tumor type before imaging. We explored possible associations between the detectability trends with tumor characteristics, and no significant correlations were found when we performed a multivariate analysis of tumor size, grade, hormone receptor status (HER2/neu, estrogen, progesterone), and Ki-67. It was expected that the groups would separate based on Ki-67, and the observed lack of correlation could be a result of the low sample size. The correlation between the biology and kinetic parameters may also have been obscured by the small tumor size and lack of partial volume correction. The link between biology and imaging could become clearer by testing delayed imaging in a patient study, requiring that patients return for a delayed scan after their scheduled clinical scan.

Recommending a patient-specific imaging time may ultimately be unfeasible or impractical. By better understanding the trade-offs with imaging time, a single time point based on the average response for a group of patients could be recommended. Group 2 showed that, even for patients who do not benefit from delayed imaging, tumor detectability may only slightly decrease with increased postinjection imaging time. Furthermore, the data acquisition time could be lengthened to compensate for the increased noise while taking advantage of the increased contrast at later time points. Modern 3D PET systems and time-of-flight technology could also reduce the impact of noise. Finally, although we focused on single time point imaging, which requires minimal changes to the current workflow and would be more feasible to implement clinically, it may be that dynamic or multiple time point imaging could help by providing the extra uptake information.

Our study had some limitations. Patient data were limited to 60-minute dynamic scans for 23 patients with low- to intermediate-grade tumors and favorable (ER+/HER2−) subtypes of breast cancer that may or may not be similar to other tumor types and subtypes. All data were from readily detectable tumors, so assumptions were made to adjust the tumors toward the limit of detectability. We do not intend to imply that the same tumor would have had the same uptake characteristics at a smaller size but rather that a different tumor could have these uptake characteristics at this size. The 2-tissue compartment kinetic model simplifies the biological processes and assumes homogeneity in the compartments (2). Detectability performance was estimated using a mathematical model observer. Although the CHO model was previously validated against human observers (30), this study evaluated a different set of images.

There is the question of FDG dephosphorylase activity and when *k*_4_ can be assumed to be negligible. If FDG phosphorylation has only minimal reversibility, it can be adequately modeled as irreversible for short-duration studies, and the tumor uptake will increase for many hours. In this study, if the acquired 60 minutes of data showed increasing FDG uptake in the tumor, the synthetic curves continued to show increasing uptake until plateauing at much later times. This result agrees with that of other studies of breast and other cancers (4–6, 8, 9) that showed that the tumor FDG concentration continues to increase for many hours, indicating that *k*_4_ is indeed small. For this reason, it is not uncommon for studies to assume that *k*_4_ = 0. For example, Hamberg et al. (7) acquired non-small cell lung cancer data for up to 90 minutes and then assumed that *k*_4_ = 0 when extrapolating to infinity. However, both Lodge et al. (4) and Spence et al. (5) acquired data for up to 6 and 8 hours and reported nonzero values of *k*_4_ in brain (gliomas and gray matter) and soft tissue masses (benign and malignant), respectively. Lodge et al. showed that dephosphorylation becomes evident in soft-tissue sarcoma TACs 4 hours after injection, and Spence et al. showed a decrease in brain tumor between 3.5 and 5 hours. In the case of brain tissue, it has been shown that the assumption of a nonreversible model is valid for up to only 2 hours after injection (3, 24). Therefore, it was important to consider the possible effects of *k*_4_ > 0 in this study.

Most dynamic studies are conducted for 60 minutes, as were the studies from which our breast cancer patient data were derived. Lucignani et al. (24) showed that a parameter estimation using the reversible model is inaccurate when using experimental data from less than 2 hours. The estimation of *k*_**4**_ is difficult from only 60 minutes of dynamic data because the effects of a small *k*_**4**_ and initially small FDG-6-P pool size do not significantly affect the uptake curves over that timeframe. Rather than estimating *k*_4_ from measured data, we decided to fix the dephosphorylation rate constant for 3 values larger than 0 based on values published in the literature. Unfortunately, only a small amount of data are available on *k*_**4**_ estimates for cancer and even less so for breast cancer specifically. Krak et al. (21) reported an average value of *k*_**4**_ = 0.003 ± 0.022 min^−1^ in locally advanced breast cancer. Tseng et al. (25) also estimated a much higher value in locally advanced breast cancer of 0.014 ± 0.012 min^−1^. The large uncertainties attest to the difficulties of estimating *k*_4_ from only 60 minutes of measured data. We believe that the *k*_4_ values we simulated spanned a range of plausible values and accounted for the effect of FDG dephosphorylation at later times without making assumptions about the magnitude of the parameter.

Using tumor properties from breast cancer FDG studies, we showed that delayed imaging at 2 to 3 hours after radiotracer injection may improve tumor detectability in some patients and minimally compromise overall tumor detection in others. We used TACs that captured the biology of the tumor to simulate realistic PET images in a virtual clinical trial. Tumor detectability depended on the tumor and normal TACs and the trade-off between increasing contrast and increasing noise as a function of time post radiotracer injection. At up to 4 hours after injection, the rate of FDG that leaves the tumor either by dephosphorylation or other pathways may be relevant to assessments of FDG uptake. This method of a virtual clinical trial can be used to guide future clinical trials in patients to further evaluate the impact of imaging time and other parameters on tumor detection, response to therapy, and outcome measures.
